# The relationship between emotional exhaustion and job embeddedness in nurses: the mediating role of decent work perception and the moderating role of job crafting

**DOI:** 10.3389/fpsyg.2025.1678739

**Published:** 2025-11-06

**Authors:** Dinuo Xin, Dina Xin, Meirong Bian, Wanling Li, Jinyan Niu

**Affiliations:** 1 Department of Nursing, Shanxi Bethune Hospital, Shanxi Academy of Medical Sciences, Third Hospital of Shanxi Medical University, Tongji Shanxi Hospital, Taiyuan, Shanxi, China; 2 Third Hospital of Shanxi Medical University, Shanxi Bethune Hospital, Shanxi Academy of Medical Sciences, Tongji Shanxi Hospital, Taiyuan, Shanxi, China; 3 Department of Nursing, Tongji Hospital, Tongji Medical College, Huazhong University of Science and Technology, Wuhan, Hubei, China

**Keywords:** nurses, emotional exhaustion, job embeddedness, decent work, job crafting cross-sectional studies

## Abstract

**Objectives:**

Nurse turnover is a global challenge facing healthcare systems, severely impacting the stability of nursing teams and the quality of care. Job embeddedness is a key predictor of nurse retention. This study aims to explore the relationship between nurses’ emotional exhaustion and job embeddedness and to analyze the mediating and moderating roles of decent work perception and job crafting in this relationship.

**Methods:**

This was a multicenter cross-sectional study. From February to March 2025, an online questionnaire was administered to 653 nurses from three general hospitals in Shanxi Province, China, using convenience sampling. The questionnaire consisted of the Demographic Information Questionnaire, Emotional Exhaustion Scale, Decent Work Perception Scale, Job Crafting Scale, and Job Embeddedness Scale. The mediating and moderating effects were tested via the PROCESS Macro 4.1 (Model 4 and Model 5) of SPSS 27.0.

**Results:**

There was a significant negative correlation between nurses’ emotional exhaustion and job embeddedness (*p* < 0.001). Decent work perception played a partial mediating role between emotional exhaustion and job embeddedness, accounting for 44.1% of the total effect. In addition, job crafting positively moderated the effect of emotional exhaustion on job embeddedness.

**Conclusion:**

Based on the results of this study, nursing managers should develop and adopt comprehensive and effective intervention strategies to reduce nurses’ emotional exhaustion, stimulate their level of decent work perception and job crafting to improve their job embeddedness, and ultimately stabilize the nursing workforce.

## 1 Introduction

With the evolution of healthcare models and the increasing trend of population aging, nurses, as the core force of the healthcare system, play an irreplaceable role in achieving the goals of comprehensive health coverage and sustainable development (Lesnik and Hauser-Oppelmayer, 2025). However, the global shortage of nurses is becoming increasingly acute for a variety of reasons, including COVID-19 (The Lancet, 2023). The World Health Organization (WHO) has reported that by 2030, there is expected to be a huge global shortage of 5.7 million nurses, with the total number of nurses falling far short of the demand for healthcare services (Catton, 2020). A major reason for the shortage of nurses is the high turnover rate of nurses. A meta-analysis of 14 studies from 21 countries showed that the global rate of nurses leaving the profession ranged from 8 to 36.6% (Ren et al., 2024). Similarly, another systematic review and meta-analysis showed that the estimated overall intent to leave the profession among nurses during the COVID-19 pandemic was 31.7% (Ulupınar and Erden, 2024). The departure of nurses exacerbates the shortage of nursing human resources, directly increases the workload of the remaining nurses, leading to a vicious cycle of declining job satisfaction and nurse turnover, which has a potential impact on the quality of nursing services and patient safety (Liang and Wang, 2025; Nam et al., 2025).

There has been a great deal of prior research on nurses’ intention to leave their jobs, but the reasons for leaving have proven difficult to determine (Sumner et al., 2025; Xu et al., 2025). Recent research has focused on exploring nurses’ intentions to stay in their jobs, that is, nurses’ desire to remain in their current job rather than seek other employment opportunities (Li et al., 2025; Yue et al., 2024). In this context, job embeddedness of nurses is of particular importance. The concept of job embeddedness, introduced by Mitchell in 2001, refers to the closeness of the network of relationships between an individual and all work-related situations inside and outside the organization, which is crucial for enhancing employees’ career loyalty and retention intentions (Holtom and O'Neill, 2004). Several studies have shown that job embeddedness is the set of all positive factors that influence employees to remain in their jobs, providing a whole new approach to thinking about predicting employee retention behavior (Chen et al., 2024; Wang H. et al., 2025). For nurses, job embeddedness can strengthen the connection between nurses and the organization, improve nurses’ sense of belonging and identification with the organization, and increase their work commitment and performance, thus guaranteeing the stability and improvement of the quality of healthcare services (Akinwande et al., 2025; Song J. et al., 2024). Therefore, exploring the influencing factors of nurses’ work embeddedness and its potential influencing mechanisms, as well as attracting and retaining qualified nurses, is of great significance for nursing managers to take proactive measures to reduce nurse turnover and ensure the stability of human resources.

## 2 Literature review and research framework

### 2.1 Emotional exhaustion and job embeddedness

Given the importance of enhancing job embeddedness, it is necessary to identify key factors that may undermine it. Among these, emotional exhaustion stands as one of the most serious threats. Emotional exhaustion is a state of exhaustion that occurs when an individual’s psychological and emotional resources are depleted, and is an important component of burnout (Attia et al., 2025). Previous studies have shown that due to the complexity of nursing work and the particularity of service recipients, nurses often face heavy workloads, high-intensity work pressure, and frequent emotional labor in their work (Cui et al., 2022; Wu et al., 2025). These factors collectively contribute to the occurrence of emotional exhaustion. A recent systematic review indicated that the prevalence of emotional exhaustion among nurses worldwide is 33.45% (Getie et al., 2025). This rate is slightly higher than the 28.9% prevalence of emotional exhaustion reported among a broader group of healthcare workers (including nurses, doctors, and other medical personnel), highlighting the more serious issue of emotional exhaustion faced by nurses (Mohamed et al., 2025). There is preliminary evidence linking this pervasive state of exhaustion to retention outcomes. For instance, a study by Dukhaykh (2023) confirmed a significant negative correlation between emotional exhaustion and job embeddedness in nurses. To theoretically support this relationship, this study draws upon the Conservation of Resources (COR) theory. This theory posits that individuals strive to acquire, retain, and protect their valuable resources—such as energy, time, and emotional capacity, and that prolonged resource depletion without any recovery or replenishment could have dual negative consequences on both individuals and the organizations (Hobfoll, 1989). According to COR theory, emotional exhaustion signifies a severe depletion of an individual’s critical psychological and physical resources. This state of resource depletion prompts individuals to adopt defensive strategies to conserve remaining resources, a process that often leads to psychological and behavioral withdrawal in their work. In addition, from the perspective of job embeddedness theory, maintaining a high level of job embeddedness requires the continuous investment of personal resources such as energy and emotions. Consequently, nurses experiencing emotional exhaustion become less capable of sustaining organizational commitment, loyalty, and work engagement as their resource reserves gradually deplete, leading to a decline in their job embeddedness (Atalla et al., 2025). Based on these considerations, the following hypothesis is proposed:

*H1:* Nurses' emotional exhaustion is negatively related to job embeddedness.

### 2.2 The mediating role of decent work perception

To elucidate the mechanism through which emotional exhaustion impairs job embeddedness, this study introduces decent work perception as a potential mediator. The concept of “decent work,” pioneered by the International Labor Organization, referring to the principle that all workers can have decent and dignified work on the basis of equality, freedom, safety and dignity, with the core elements of guaranteeing the individual’s employment rights, employment equity, social security and social dialogue (Xue et al., 2025). At the individual level, the decent work perception refers to an individual’s subjective perception regarding the value and dignity of their work, which is mainly reflected in the degree of recognition of the job position, work atmosphere and career development, job rewards, and professional recognition (Othman et al., 2025). Due to the nature of their profession, nursing staff are gradually encountering unseemly and unfair situations at work, and the physical, psychological and social risks they face are also constantly increasing (Zheng et al., 2025; Zhong et al., 2024). We propose that emotional exhaustion is a key driver that erodes nurses’ decent work perception. Empirically, research has indicated a negative correlation between job burnout and decent work perception among nurses (Zhang et al., 2024b). Theoretically, this relationship can be understood through the lens of Affective Events Theory (AET). The AET posits that specific work events can trigger emotional responses in individuals, and these responses in turn shape their attitudes towards and cognitive evaluations of the work environment, thereby influencing subsequent behavioral responses (Tu et al., 2025). In the AET framework, emotional exhaustion can be regarded as a persistent negative emotional state triggered by recurring work-related events. The accumulation of this state may diminish individuals’ sense of identity and value derived from their profession, negatively impacting their perception of decent work (Yang et al., 2025). Therefore, we propose the following hypothesis:

*H2:* Emotional exhaustion is negatively related to decent work perception.

Several studies have confirmed that guaranteeing decent work for nurses can promote many positive work outcomes (Xue et al., 2024a; Sönmez et al., 2023). A study of new nurses found that nurses’ decent work perception was positively correlated with job embeddedness (Song Z. et al., 2024). We explain this relationship through Social Exchange Theory (SET). SET posits that the relationship between an organization and its employees is based on reciprocity. It is based on the principle that if one party performs a beneficial behavior towards the other party, it creates an obligation on the party receiving the behavior that must reciprocate (Ahmad et al., 2022). According to SET, when nurses perceive a favorable working environment, satisfactory salary and benefits, and smooth career development opportunities, it indicates that they have a high recognition of the value of the job, and thus are willing to continue to establish high-quality reciprocal exchange relationships with the organization (El-Gazar and Zoromba, 2025). During this process, in return for the benefits provided by the organization, nurses are more willing to closely integrate with the organization, dedicating more time and effort to their roles. This strengthens their structural and psychological ties to both their work and the organization, manifesting as higher levels of job embeddedness (Zhang et al., 2023). Based on this, we hypothesize:

*H3:* The decent work perception by nurses is positively correlated with job embeddedness, and it plays a mediating role in the relationship between emotional exhaustion and job embeddedness.

### 2.3 The moderating effect of job crafting

Beyond identifying the mediating mechanism, it is equally crucial to explore potential factors that could buffer the detrimental effect of emotional exhaustion. In this study, we propose job crafting as such a moderating factor. Job crafting refers to the process in which employees actively adjust and optimize their work tasks, relationships, and cognition, thereby achieving a better match between individuals and their work (Wang et al., 2024). The Job Demands-Resources (JD-R) model suggests that job demands (such as job stress, workload) and job resources (such as autonomy, support) in the work environment are key factors affecting employees’ psychological well-being and job performance (Zeng et al., 2025). When individuals face high job demands such as emotional exhaustion without sufficient job resources, they are prone to sustained depletion, leading to negative outcomes like work alienation and decreased performance (Fan et al., 2025; Xin et al., 2024). Job crafting, as a positive job resource, enables nurses to redesign responsibilities, regulate interpersonal interactions, and actively reconstruct work meaning, thereby effectively buffering psychological attrition caused by emotional exhaustion (Guo et al., 2024). Specifically, nurses with high job crafting are more likely to mobilize personal initiative in stressful situations. By enhancing task mastery, seeking social support, and reshaping perceptions of professional value, they maintain organizational identification and connection, thereby mitigating the erosion of work embeddedness caused by emotional exhaustion (Yun et al., 2024; Zhang Y. et al., 2024; Zohourparvaz and Vagharseyyedin, 2023). Conversely, nurses lacking the capacity for job crafting are more likely to become passive in the face of emotional exhaustion, and have trouble maintaining their level of embeddedness in their work (El-Ashry et al., 2025). Based on the above statements, the following hypothesis is proposed:

*H4:* Job crafting moderates the relationship between emotional exhaustion and job embeddedness.

In summary, while there exists a certain degree of association between emotional exhaustion, decent work perception, job crafting, and job embeddedness, the underlying mechanisms linking these factors have yet to be fully explored. This study ultimately constructed a comprehensive framework based on JD-R model. Within this framework, decent work perception is regarded as a crucial environmental resource, job crafting as a positive personal resource, and emotional exhaustion as a job demand that depletes job resources. Therefore, this study aims to examine a moderated mediation effect model with emotional exhaustion as the independent variable, job embeddedness as the dependent variable, decent work perception as the mediating variable, and job crafting as the moderating variable. Ultimately, the overall goal of this study is to provide theoretical and practical guidance for promoting nurses’ job embeddedness and sustainable development, enhancing the stability of the nursing workforce, and offering new insights for nursing managers.

## 3 Methods

### 3.1 Design

This study used a multicenter cross-sectional study design and strictly adhered to the Strengthening the Reporting of Observational Studies in Epidemiology (STROBE) statement.

### 3.2 Setting and samples

From February to March 2025, nurses from three general hospitals in Shanxi Province, China, were selected for the survey using a convenience sampling method. The inclusion criteria for participants were as follows: (1) registered nurses with a nursing license; and (2) informed consent and willingness to participate in the study. Exclusion criteria included (1) nurses who were on vacation during the survey period; (2) internship nurses; and (3) nurses in advanced training. According to Kendall’s principle of sample size estimation, the sample size is 10–20 times the number of independent variables (Fang et al., 2025). This study contains 21 independent variables, so the required sample size is 21 × (10–20) = (210–420). Considering 20% invalid questionnaires, a sample size of 263 to 525 nurses was required. A total of 672 questionnaires were finally returned, of which 653 were valid, giving an effective return rate of 97.2%.

### 3.3 Ethical considerations

This study was approved by the Medical Ethics Committee of Shanxi Bethune Hospital (No. YXLL-2023290). All participants followed the principles of informed consent and voluntary participation in the study. All data were used only for this study and were kept strictly confidential.

### 3.4 Instruments

#### 3.4.1 The demographic questionnaire

The demographic information was designed by the researcher according to the purpose of the study and included gender, age, education level, professional title, working experience, department, marital status, and monthly income.

#### 3.4.2 Emotional exhaustion scale

In this study, emotional exhaustion was measured using the emotional exhaustion section of the Burnout Scale developed by Maslach and Jackson (1981). The scale consists of nine items, such as “Work makes me emotionally drained,” which are answered on a 7-point scale (“1” = never, “7” = every day). The total scale score ranges from 9 to 63, with higher scores representing greater emotional exhaustion. The Cronbach’s *α* coefficient for this scale in this study was 0.939.

#### 3.4.3 Decent work perception scale

This scale was developed by Mao et al. (2014) to assess the degree of decent work perceived by employees. The scale consists of 16 entries in 5 dimensions: job position, career development, professional recognition, work atmosphere, and job reward. The scale was rated on a 5-point Likert scale from “1” (strongly disagree) to “5” (strongly agree), with a total score from 16 to 80. Higher scores indicate higher levels of perceived decent work. The Cronbach’s *α* coefficient for this scale in this study was 0.918.

#### 3.4.4 Job embeddedness scale

This scale was developed by Crossley et al. (2007). Mei et al. (2014) translated and revised it. It consists of 7 single-dimensional items. The scale used a Likert 5-point scale, scoring 1 to 5 points from “strongly disagree” to “strongly agree.” Items 4 and 6 are reverse-scored. The total score ranges from 7 to 35, with a higher score indicating a higher level of job embeddedness. In this study, the Cronbach’s α coefficient of this scale was 0.897.

#### 3.4.5 Job crafting scale

This scale was developed by Dvorak (2014) and was translated into Chinese by Zhu (2019). It was used to evaluate the current situation of nurses’ job crafting. The scale consists of 3 dimensions: relationship crafting, task crafting, and cognitive crafting, with a total of 21 items. It uses the Likert 5-point rating method, scoring from “strongly disagree” to “strongly agree,” with 1 to 5 points, respectively. The total score ranges from 21 to 105, and the higher the score, the better the situation of nurses’ job crafting. In this study, the Cronbach’s α coefficient of this scale was 0.966.

### 3.5 Data collection

This study was conducted online through the Questionnaire Star website. The researcher explained the purpose and significance of the study to the nursing leaders in each hospital prior to the survey and obtained their consent and support for the study. Subsequently, the electronic questionnaire was distributed by the nursing department leaders to the WeChat group of nurses in each department. The instructions emphasized the purpose of the survey, the considerations for completing it, and important notes and explanations. Participants could only proceed to the next step after reading and agreeing to the informed consent form, and could withdraw from the survey at any time during the process. To ensure the validity of the questionnaire and to prevent repetition and omission, all questions were set as mandatory and could only be filled out once by an IP. Finally, invalid questionnaires that were regularly answered, illogical, or too short to fill out were eliminated.

### 3.6 Data analysis

The study was statistically analyzed using SPSS 27.0. First, descriptive analysis (mean, standard deviation, number of cases, percentage) was used to evaluate participants’ demographic characteristics and scores on the main variables. Second, Pearson correlation analysis was used to evaluate the correlations between the main variables. Finally, Model 4 in PROCESS Macro 4.1 was used to evaluate the mediating role of decent work perception for nurses between emotional exhaustion and job embeddedness, and Model 5 was used to evaluate the moderating role of job crafting between emotional exhaustion and job embeddedness. To further validate the moderating role of job crafting, a simple slope test was used to assess the relationship between emotional exhaustion and job embeddedness at different levels of job crafting. Indirect effects of mediating effects were assessed using a bootstrap method with 5000 samples, and 95% confidence intervals (CIs) excluding zero indicated that these effects were significant. All statistical analyses were two-sided, and *p*-values less than 0.05 were considered statistically significant.

## 4 Results

### 4.1 Common method bias test

As the data in this study were self-reported, there may be potential common method bias. Therefore, we performed Harman’s one-way test on the data to check for common method bias. The results showed that there were eight factors with eigenvalues greater than 1, and the first common factor explained 32.9% of the total variance, which was below the 40% critical threshold. Thus, this study was not significantly affected by common method bias.

### 4.2 Demographic characteristics of participants

Of the 653 participants, 627 (96.0%) were female, and 341 (52.2%) were between the ages of 30 and 39 years old. 626 (95.9%) of the participants held a bachelor’s degree, and 344 (52.7%) held an intermediate title. In addition, 451 participants (69.1%) were married and 523 (80.1%) were on contract. The majority of the participants had 11–15 years of experience, representing 40.9% of the sample size. Detailed general information of the participants is presented in Table 1.

**Table 1:** Demographic characteristics of nurses (*N* = 653).

Characteristics	Categories	*N*	%
Gender	Male	26	4.0
	Female	627	96.0
Age	≤29	229	35.1
	30 ~ 39	341	52.2
	≥40	83	12.7
Education level	Junior college	6	0.9
	Bachelor	626	95.9
	Master or above	21	3.2
Professional title	Junior	298	45.6
	Intermediate	344	52.7
	Senior	11	1.7
Marital status	Married	451	69.1
	Unmarried	198	30.3
	Other	4	0.6
Employment methods	Contact system	523	80.1
	Permanent	130	19.9
Work experience (years)	≤5	226	34.6
	6–10	61	9.3
	11–15	267	40.9
	16–20	66	10.1
	>20	33	5.1
Work department	Internal medicine	228	34.9
	Surgical	169	25.9
	Gynecology and obstetrics	51	7.8
	Pediatric	40	6.1
	Oncology	36	5.5
	ICU or emergency	11	1.7
	Operating room	43	6.6
	Outpatient and technical departments	49	7.5
	Other	26	4.0
Monthly income (RMB)	≤5000	77	11.8
	5001–9000	415	63.6
	9001–13000	137	21.0
	>13000	24	3.7
Weekly working time (hours)	≤40	83	12.7
	41–50	450	68.9
	51–60	93	14.2
	>60	27	4.1
Average number of night shifts per month	None	137	21.0
	1–3 times	80	12.3
	4–6 times	203	31.1
	7–9 times	194	29.7
	>9 times	39	6.0

M, mean; SD, standard deviation.

### 4.3 Descriptive statistics and correlation analysis of variables

The results of the means, standard deviations, and correlation coefficients of the variables of this study are shown in Table 2. The mean score of nurses’ emotional exhaustion was 31.15 ± 11.47, the mean score of decent work perception was 53.40 ± 10.44, the mean score of job embeddedness was 24.06 ± 4.99, and the mean score of job crafting was 72.06 ± 13.75. The Pearson correlation analysis revealed that emotional exhaustion was negatively correlated with the decent work perception (*r* = −0.455, *p* < 0.001), job embeddedness (*r* = −0.437, *p* < 0.001), and job crafting (*r* = −0.302, *p* < 0.001). The decent work perception was positively correlated with job embeddedness (*r* = 0.535, *p* < 0.001) and job crafting (*r* = 0.363, *p* < 0.001). Moreover, job crafting was positively correlated with job embeddedness (*r* = 0.345, *p* < 0.001).

**Table 2:** Descriptive statistics and correlation analysis.

Variables	M ± SD	1	2	3	4
1. Emotional exhaustion	31.15 ± 11.47	–			
2. Decent work perception	53.40 ± 10.44	−0.455***	–		
3. Job embeddedness	24.06 ± 4.99	−0.437***	0.535***	–	
4. Job crafting	72.06 ± 13.75	−0.302***	0.363***	0.345***	–

M, mean; SD, standard deviation. *** *p* < 0.001.

### 4.4 Mediating effect analysis

To assess the mediating effect of nurses’ decent work perception between emotional exhaustion and job embeddedness, Model 4 in PROCESS was utilized. The results showed that emotional exhaustion had a significant negative effect on decent work perception (*β* = −0.233, *t* = −13.039) and job embeddedness (*β* = −0.245, *t* = −12.409). After using decent work perception as a mediating variable, the direct effect of emotional exhaustion on job embeddedness remained significant (*β* = −0.137, *t* = −6.797), and decent work perception was significantly positively correlated with job embeddedness (*β* = 0.464, *t* = 11.800) (Table 3). Bootstrap analyses showed that emotional exhaustion indirectly affects job embeddedness through the decent work perception, with a 95%CI that did not contain 0 (indirect effect = −0.108, 95%CI = (−0.149, −0.074)), indicating a statistically significant mediating effect. Furthermore, the mediating effect accounted for 44.1% of the total effect (Table 4).

**Table 3:** Testing for the mediation effect.

Predictors	Model 1 (job embeddedness)		Model 2 (decent work perception)		Model 3 (job embeddedness)	
	*β*	*t*	*β*	*t*	*β*	*t*
Emotional exhaustion	−0.245	−12.409***	−0.233	−13.039***	−0.137	−6.797***
Decent work perception	–	–	–	–	0.464	11.800***
*R* ^2^	0.191		0.207		0.334	
*F*	153.993***		170.015***		162.960***	

*** *p* < 0.001.

**Table 4:** The total, direct, and indirect effects of the mediation model.

Path	*β*	*SE*	*LLCI*	*ULCI*	%
Total effects	−0.245	0.020	−0.284	−0.206	100.0
Direct effects	−0.137	0.020	−0.176	−0.097	55.9
Indirect effects	−0.108	0.019	−0.149	−0.074	44.1

### 4.5 Moderating effects analysis

**Figure 1:**
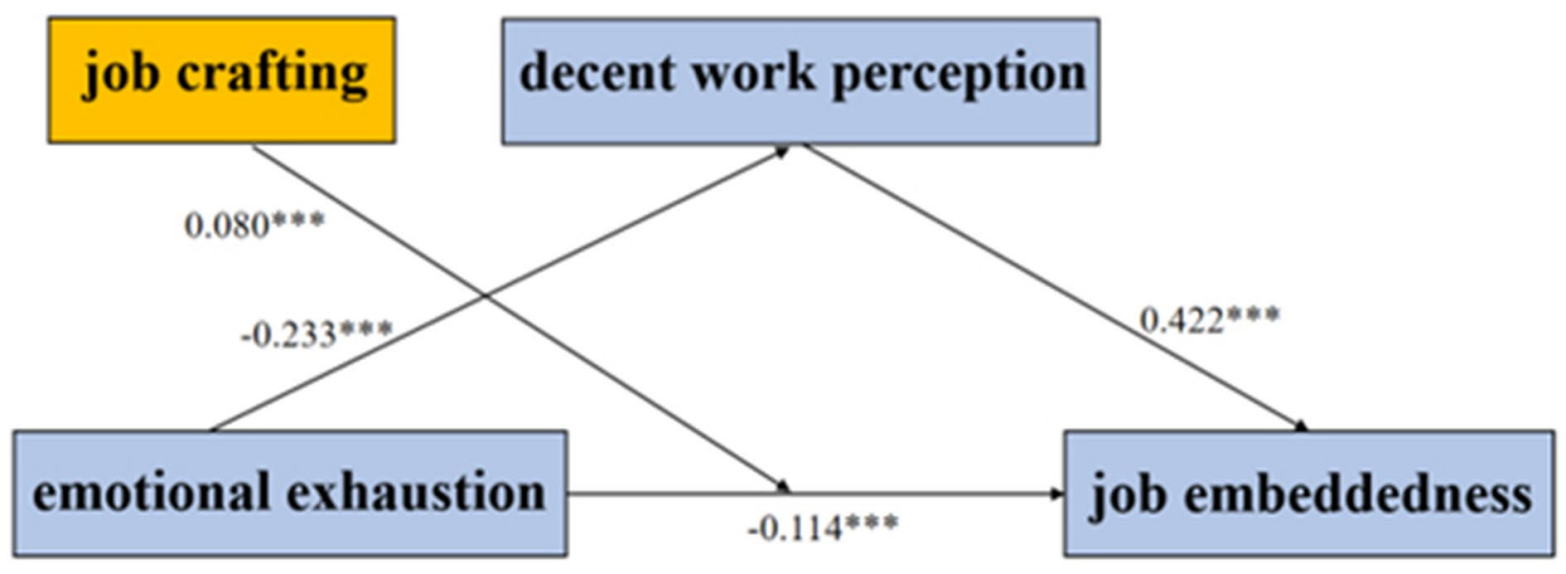
Path coefficient of the model.

Model 5 in PROCESS was applied to examine the moderating effects of job crafting. The results showed that emotional exhaustion significantly negatively predicted decent work perception (*β* = −0.233, *t* = −13.039) and job embeddedness (*β* = −0.114, *t* = −5.701). Decent work perception (*β* = 0.442, *t* = 10.558) and job crafting (*β* = 0.144, *t* = 3.873) also significantly positively predicted job embeddedness. Additionally, the interaction term between emotional exhaustion and job crafting significantly positively predicted job embeddedness (*β* = 0.080, *t* = 3.444), indicating that job crafting positively moderated the relationship between emotional exhaustion and job embeddedness (Table 5; Figure 1).

**Table 5:** Coefficients for the tested moderating model.

Predictors	Model 1 (job embeddedness)		Model 2 (decent work perception)	
	*β*	*t*	*β*	*t*
Emotional exhaustion	−0.114	−5.701***	−0.233	−13.039***
Decent work perception	0.422	10.558***		
Job crafting	0.144	3.873***		
Emotional exhaustion × job crafting	0.080	3.444***		
*R* ^2^	0.362		0.207	
*F*	91.891***		170.015***	

× Represents the interaction of emotional exhaustion and job crafting. *** *p* < 0.001.

**Figure 2:**
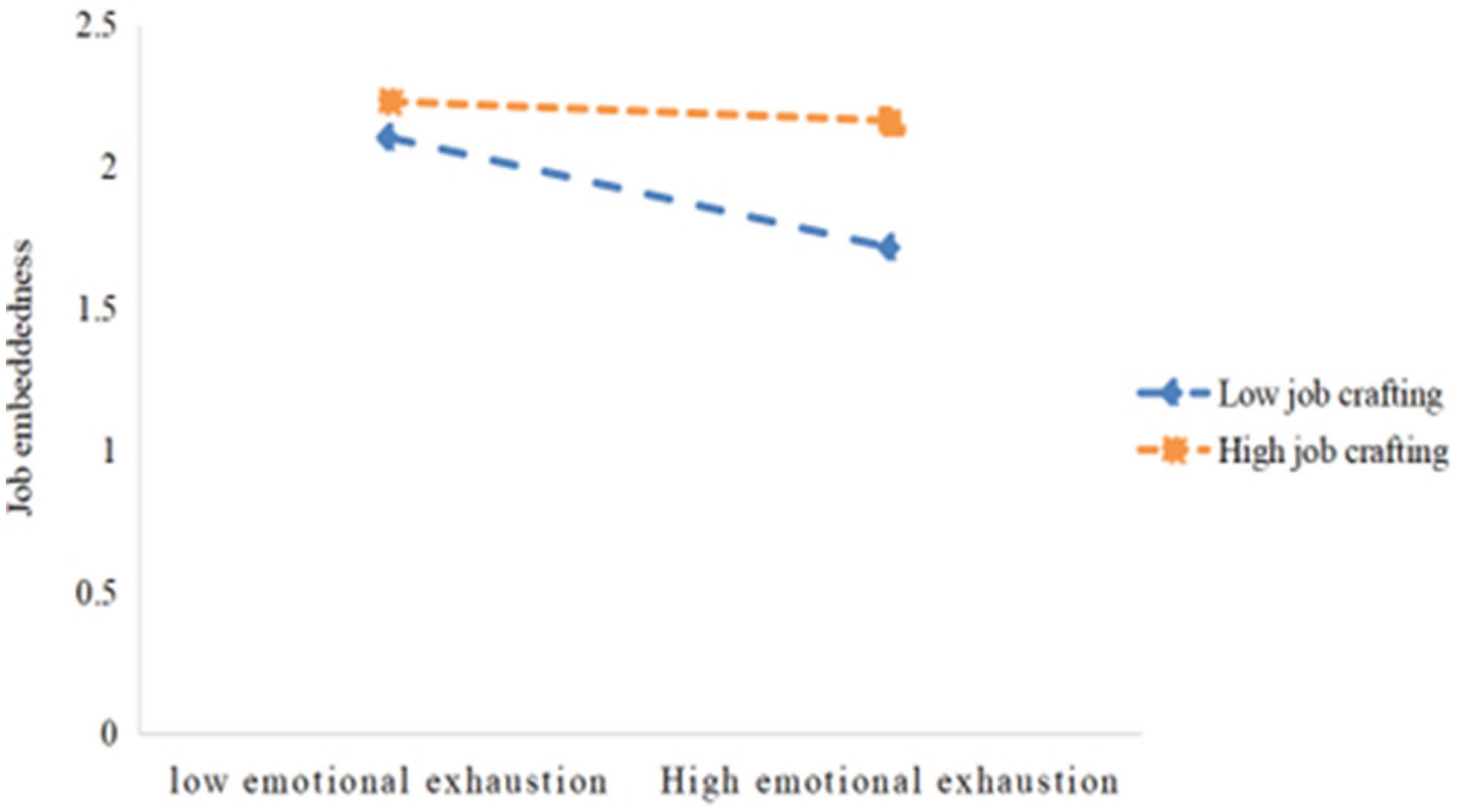
Moderating effect diagram of job crafting.

To better account for this moderating effect, we conducted a simple slope analysis to categorize participants into low and high job crafting groups based on standard deviations below or above the mean. As shown in Figure 2 and Table 6, at lower levels of job crafting, emotional exhaustion was negatively associated with job embeddedness (*β* = −0.167, *p* < 0.001). However, at higher levels of job crafting, emotional exhaustion showed a weaker negative association with job embeddedness (*β* = −0.063, *p* = 0.019). These results suggest that the negative correlation between emotional exhaustion and job embeddedness becomes weaker with increasing job crafting, namely, job crafting plays a positive moderating role between emotional exhaustion and job embeddedness.

**Table 6:** Conditional effects of emotional exhaustion on job embeddedness at different levels of job crafting.

Job crafting	*β*	*SE*	*t*	*p*	*LLCI*	*ULCI*
M-1SD	−0.167	0.024	−7.054	<0.001	−0.214	−0.121
M	−0.115	0.020	−5.701	<0.001	−0.155	−0.075
M + 1SD	−0.063	0.027	−2.346	0.019	−0.115	−0.010

## 5 Discussion

Based on prior research and theory, this study examined the relationship between nurses’ emotional exhaustion and job embeddedness, as well as the role of decent work perception and job crafting in the relationship. The findings revealed a direct negative association between nurses’ emotional exhaustion and their job embeddedness. Furthermore, decent work perception was identified as a significant mediator in this relationship. Moreover, job crafting buffered the negative relationship between emotional exhaustion and job embeddedness, playing a positive moderating role. These findings deepen our understanding of the mechanisms by which nurses’ emotional exhaustion affects job embeddedness and provide a valuable theoretical and practical basis for nursing managers to develop targeted interventions to promote nurses’ job embeddedness.

### 5.1 The relationship between emotional exhaustion and job embeddedness in nurses

This study found a negative correlation between nurses’ emotional exhaustion and job embeddedness, which supports H1. This finding is consistent with previous research, which suggests that the level of emotional exhaustion is a key factor influencing nurses’ job embeddedness (Dukhaykh, 2023). Meanwhile, the result fits with the COR theory. For a long time, nurses have been undertaking extremely demanding caregiving tasks, and they also face issues such as occupational stress, work–family conflicts, and an imbalance between efforts and rewards. These problems can constantly deplete their physical and psychological resources, which could potentially contribute to emotional exhaustion once the resources are depleted to a certain point (Tan et al., 2025). When nurses perceive a depletion of their emotional resources and fail to receive timely replenishment, they might develop negative feelings and attitudes towards their work, showing symptoms such as emotional exhaustion and loss of energy, which could be linked to decreased organizational commitment and retention intentions (Zhang X. et al., 2025). Job embeddedness, as a comprehensive measure of the strength of an individual’s connection to the organization, is dependent on emotional commitment, perceived organizational support, and resource exchange processes. However, emotional exhaustion significantly associated with diminished nurses’ motivation to maintain organizational connectedness, which correlates with decreased levels of job embeddedness (Zhang Y. et al., 2025).

Therefore, in order to reduce nurses’ emotional exhaustion, nursing managers should implement a variety of strategies. First, they should focus on humanistic care by adopting a combination of flexible scheduling and reasonable rest rotation to ensure that nurses get enough rest in their spare time, maintain work-life balance, and reduce emotional resource depletion due to overwork (Su et al., 2023). Secondly, a regular communication and feedback mechanism should be established, including nurse suggestion boxes and regular symposiums, which are used to promptly listen to and respond to their work demands, helping them alleviate the psychological pressure brought by work (Qiu et al., 2025). Finally, the department of nursing should introduce professional psychological intervention resources, regularly carry out stress management and individual psychological counseling services to help nurses master the skills of emotional regulation; at the same time, managers should encourage mutual assistance and collaboration among colleagues, and strengthen the emotional connection through team-building activities (Liu et al., 2025).

### 5.2 The mediating role of decent work perception

This study found that decent work perception partially mediates the relationship between nurses’ emotional exhaustion and job embeddedness. This result is in line with previous studies (Fang et al., 2025; Zhang et al., 2024b), supporting Hypotheses 2 and 3. It is particularly noted that this study found the mediating effect of decent work perception accounted for as much as 44.1% of the total effect. This significant effect size indicates that a substantial portion of the negative relationship between emotional exhaustion and job embeddedness is mediated through the pathway of weakening nurses’ decent work perceptions. This finding reveals that emotional exhaustion serves as a key antecedent variable undermining nurses’ decent work perceptions, and clarifies the core mediating mechanism through which decent work perceptions influence nurses’ job embeddedness. The decent work perception refers to an individual’s subjective perception of the social value of their profession, and is a key factor in enhancing the enthusiasm of nurses and their sense of organizational belonging. Nurses with a high level of decent work perception are more likely to recognize their work, and are also better able to meet their own development needs and realize their own value through their work (Xue et al., 2024b). When nurses fall into a state of resource deprivation due to emotional exhaustion, their sense of professional efficacy and value is likely to be lower. They become disappointed with structural resources such as pay equity, promotion opportunities, and social status, and find it difficult to find positive meaning and positive feedback in their work, which is associated with a weaker perception of decent work (Feng et al., 2025). The lower perception of decent work is associated with diminished intrinsic motivation, along with weaker emotional attachment to the organization, and a lower level of job embeddedness (Song Z. et al., 2024).

These findings offer new insights and implications for practice: Rather than directly addressing emotional exhaustion, interventions that protect and enhance nurses’ perceptions of decent work may serve as an effective entry point for strengthening job embeddedness and mitigating staff turnover. Therefore, healthcare workers must recognize the importance of implementing the principle of decent work in nursing practice. This study proposes tiered implementation recommendations.

First, at the organizational management level, short-term supportive measures that can be rapidly implemented should be adopted. This includes implementing employee assistance programs to provide nurses with professional psychological counseling and stress management training; carrying out flexible scheduling systems to help nurses achieve work-life balance; and establishing appropriate recognition and incentive mechanisms to provide timely feedback on nurses’ contributions. These measures aim to rapidly replenish nurses’ psychological resources and mitigate the direct impact of emotional exhaustion (Xue et al., 2025).

Second, comprehensive systemic structural reforms must be implemented to fundamentally consolidate and enhance nurses’ perception of decent work. On one hand, nursing administrators should create a magnetic work environment, improve and optimize the salary structure and welfare protection system, establish a fair and reasonable promotion mechanism and diversified career development paths, and provide adequate training and further training opportunities according to nurses’ personal characteristics and abilities to stimulate their work motivation (Zheng et al., 2025). Simultaneously, organizational support must be strengthened by valuing nurses’ decision-making voice and sense of ownership to enhance their professional identity (Zheng et al., 2024). On the other hand, governments and society should actively guide and encourage all sectors of society to establish a correct concept of the professional status and image of nurses, and to give full understanding and respect to the nursing profession, so as to enhance the nurses’ own sense of value and mission (Cai et al., 2025). Only through this strategy combining short-term interventions with long-term structural reforms can we effectively enhance nurses’ decent work perceptions, ultimately achieving the stability and healthy development of the nursing workforce.

### 5.3 The moderating role of job crafting

Another important finding of this study was that job crafting moderated the direct association of nurses’ emotional exhaustion on job embeddedness, supporting Hypothesis 4. That is, the negative association of emotional exhaustion on job embeddedness appeared to be weaker as the level of job crafting increased. Similar findings have been reported in previous studies (Zhang et al., 2024a). Based on the J-DR Theory model, van Zyl et al. (2025) proposed that job crafting is a proactive behavior to realize the matching of job demands and resources with one’s own abilities and needs, which is a concrete expression of nurses’ search for meaning and value of their work. At the same time, job crafting may serve as a protective resource against adverse effects such as job stress and burnout. Specifically, when faced with job demands such as emotional exhaustion, nurses with high levels of job crafting might be better equipped to transform negative emotions into the motivation to enhance their work experience by flexibly adjusting their work methods, optimizing interpersonal relationships, and reconstructing the meaning of their profession (Han, 2023). This process is conceptually linked to greater workplace resilience, as well as sustained emotional commitment and person-organization fit, which in turn is associated with higher job embeddedness (Zhang H. et al., 2024). Conversely, nurses with low levels of job crafting may not engage in positive self-adjustments when faced with emotional exhaustion. They are prone to developing psychological discomfort, such as anxiety and self-doubt, and the degree of their connection with the job also weakens accordingly (He et al., 2025).

Therefore, nursing managers should authorize nurses to participate in the design and optimization of work content, allowing them to independently adjust the work link within the normative framework to enhance the sense of control over work content (Saleh et al., 2024). Secondly, targeted training should be carried out, such as through case studies, to guide nurses to learn how to reconstruct the meaning of work and optimize interpersonal collaboration (Park and Ha, 2025). Finally, managers should create an inclusive organizational atmosphere, encouraging nurses to make work improvement suggestions and give them positive feedback. At the same time, nurses’ interpersonal and clinical communication skills should be strengthened to realize the exchange of experience and mutual assistance in the workplace through relational crafting, and then enhance the level of work embeddedness (Wang Y. et al., 2025).

## 6 Limitation

Despite the significant findings of this study, there are some limitations. First, this study used convenience sampling and surveyed nurses from only three general hospitals in Shanxi Province. Furthermore, the sample consisted almost entirely of female participants with higher education levels, which limits the generalizability and extrapolation of the findings. Future studies may employ multistage stratified random sampling to expand the sample scope to different provinces and various types and levels of healthcare institutions. Additionally, they should specifically include more male nurses and nurses with lower educational attainment to increase the applicability of the findings. Secondly, the cross-sectional design limits causal inference. Although the tested mediation and moderation models were theoretically grounded, the observed relationships were correlational and could not establish causality. The longitudinal or experimental design is necessary in the future to better understand the dynamic mechanisms among the variables. Thirdly, the data in this study relied on nurses’ self-subjective reports, which may be subject to recall bias and social desirability bias. Although this study employed Harman’s single-factor test, this alone is insufficient to completely rule out the influence of common method bias. Future studies could incorporate data from other objective evaluations or third-party reports to improve the reliability of the results. The findings of this study may be influenced by unique cultural factors, such as local work norms, values, and organizational practices. Therefore, the conclusions drawn may have limitations when applied to nursing populations living in culturally distinct environments. Future research is needed to examine the cross-cultural applicability of these findings.

## 7 Conclusion

Nurses are an important part of the healthcare system, and a stable nursing team not only helps to improve the quality of nursing services but also promotes the improvement of the overall healthcare level of the hospital. Therefore, it is particularly important to enhance nurses’ job embeddedness and reduce the turnover rate of nurses. This study developed a mediated moderation model from the perspective of clinical nurses to explore the processes and mechanisms by which emotional exhaustion affects nurses’ job embeddedness. The results showed that decent work perception partially mediated the relationship between nurses’ emotional exhaustion and job embeddedness, and job crafting positively moderated the negative impact of emotional exhaustion on job embeddedness. Nursing managers should adopt all-around and effective interventions to improve nurses’ decent work perception and job crafting, reduce the negative impact of emotional exhaustion, so as to enhance the degree of job embeddedness and promote the stable development of the nursing workforce.

## Data Availability

The raw data supporting the conclusions of this article will be made available by the authors, without undue reservation.
